# An Observational Study of Park Attributes and Physical Activity in Neighborhood Parks of Shanghai, China

**DOI:** 10.3390/ijerph17062080

**Published:** 2020-03-20

**Authors:** Xinxin Wang, Chengzhao Wu

**Affiliations:** 1 Department of Landscape Architecture, College of Horticulture, Post-doctoral Research Station in Public Administration, Nanjing Agricultural University, Nanjing 210095, China; 2 Department of Landscape Studies, Key Lab of Ecology and Energy Saving in High-density Human Settlements, College of Architecture and Urban Planning, Tongji University, Shanghai 200092, China; wuchzhao@vip.sina.com

**Keywords:** park usage, landscape features, levels of use, behavior mapping, health benefits

## Abstract

Evidence shows that neighborhood parks provide opportunities for urban residents to participate in physical activity, but little is known about the space–behavior relationship of physical settings. This study explored the patterns of use in neighborhood parks, and focused particularly on visitors’ levels of activity supported by the specific landscape features and attributes. Behavior mapping data, including the users’ characteristics, their behaviors and activity levels, and the landscape characteristics, were obtained in three neighborhood parks of Shanghai, China. A total of 6126 park users were documented during the observations, and most of them were involved in sedentary activity. This study found that different environmental settings such as water, plaza, lawn, and architecture supported different types and levels of activity. Although more men than women visited the neighborhood parks, women were more active than men in park-based physical activity. In this Chinese sample, the findings demonstrate behavior mapping is a promising tool to measure park-based physical activity. As this study associated the levels of use with the landscape features, the results are expected to be useful in design practice for promoting regular physical activity.

## 1. Introduction

Regular physical activity is essential for maintaining health status and reducing the risk of chronic diseases such as diabetes, cardiovascular disease, and metabolic syndrome [1,2,3]. Prolonged physical inactivity can have serious health consequences. It has been estimated that inadequate levels of physical activity were responsible for 8.3% of deaths in the United States and 10.4% of deaths in Europe [4,5]. Compared to Western countries, China has experienced a higher rate of decrease in physical activity [6], and in urban China the situation was even worse—only 7.9% of the urban adults participated in moderate or vigorous physical activity (MVPA) in their leisure time (28.9% for rural adults) [7,8]. It is of special concern to promote physical activity among urban residents in China [9].

Previous research has indicated that environmental factors may influence the rates of physical activity [10,11], and living close to urban parks or other green spaces is linked to increased levels of physical activity [12,13,14,15]. Research has also found that conducting exercise in a green environment is more beneficial than doing the same exercise in an indoor environment [16]. By visiting urban parks, diverse and significant health benefits can be obtained, such as better sleep, stronger muscles, improved mood, reduced stress, and social contact [17,18].

Numerous studies have shown neighborhood parks provide ideal places for physical activity [19], emphasizing that the presence of or access to nearby nature can contribute to mental and physical health [20,21,22,23,24,25]. Compared with the large number of studies examining the influence of neighborhood-built environment on park usage [26,27], especially the proximity to a park and the surrounding communities [28,29,30], few studies have focused on the specific design features that may influence the park use and the occurrence of physical activity [31].

Evidence shows that park attributes are related to park usage, that is, a large size, the quality of facilities, organized activities, and good maintenance can promote overall park use [31,32,33]. An observational study of plaza users in San Francisco found that the microclimate conditions, such as temperature, humidity, and sunshine, influenced the behaviors of visitors [34]. Although these studies suggest that physical activities were influenced by park attributes [35], little has been learned about the specific landscape components supporting the different types of physical activity. Several recent studies have addressed participants’ visual preference for park features [36,37]. As these studies mainly focused on the perspective aspect of landscape components, the space–behavior relationship was not clear for the preferred landscape features.

Behavior mapping is an objective method for linking physical activity and outdoor design, which has been applied in studies of people’s behaviors in urban streets [38], childcare centers [39], schools [40], hospitals [41], and neighborhood open space [42]. This method allows researchers to associate the design of behavior settings with physical activity levels among participants. The validated direct observation tools such as the System for Observing Play and Recreation in Communities (SOPARC) and the System for Observing Play and Leisure Activity in Youth (SOPLAY) have been developed to obtain direct information on park users and their physical activity [40,43,44]. Using behavior mapping and geographic information system (GIS)-supported techniques, Goličnik and Ward Thompson analyzed the use patterns and spatial occupancy of three urban parks in Edinburgh and Ljubljana [45]. Using the SOPARC tool, previous research examined the conditions, user characteristics, and their physical activity in neighborhood parks of the United States at two time points [46].

The behavior mapping method is based on two theoretical perspectives. The affordance theory emphasizes the relations between perceived properties of the environment and the individual’s possibilities for action; people can perceive the use value of the environment in a direct and immediate way [47]. The concept of affordance helps the investigators to understand how the varied landscape attributes attract different types of physical activity. From an ecological perspective, Barker’s “behavior setting theory” explains how the physical environments and the patterns of behaviors are linked together; the analyzing unit “behavior setting” is characterized by activities people performed within specific time intervals and spatial boundaries [48]. In the context of neighborhood parks, behavior setting can be used to analyze people’s levels of physical activity affected by landscape environments of various attributes and qualities.

Neighborhood parks are the important components of an urban green space system, and have been highlighted as the nearest natural environment available for urban citizens [21]. Although neighborhood parks play an important role in supporting physical activity, they tend to be underutilized, especially for moderate to vigorous exercise [49]. Studies in China have assessed the spatial accessibility to parks in urbanized areas by analyzing the movement patterns of urban citizens, to value the spatial disparities in the distribution of parks and green spaces [50,51]. These studies considered spatial equity in urban green resources on a city scale, but little has been learned about how the specific landscape components within the boundaries of neighborhood parks affect the levels of use. Previous research has shown that nature-based components (e.g., vegetation) received higher perceived restorativeness and stress recovery effects than hardscape features (e.g., plaza) [52,53]. The above studies used representative photos or videotaped scenes as research materials, and no data were collected on site from the actual users, so how the landscape features and attributes influenced the behaviors of park users were understudied. In order to understand the impact of design on park usage, and how the physical environments supported the individual’s actions, research should consider the relationship between landscape settings and levels of physical activity in neighborhood parks [54].

This study used behavior mapping and GIS techniques to investigate how park visitors’ levels of physical activity, such as sedentary, walk, and MVPA, were affected by different types of behavior settings and attributes in neighborhood parks, including water, plaza, lawn, and architecture. The findings can help improve design and management of neighborhood parks in Chinese cultural settings, particularly for promoting physical activity.

## 2. Materials and Methods

### 2.1. Study Sites

**Figure 1:**
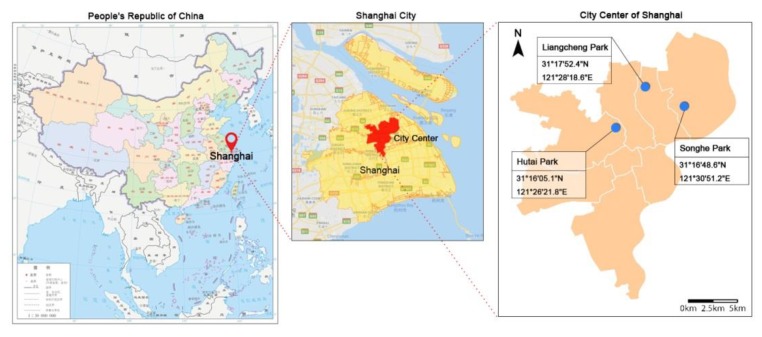
Locations of the study sites (Sources: Gov.cn [56], Google Maps [57]).

The three neighborhood parks selected for this study were Songhe Park, Liangcheng Park, and Hutai Park in Shanghai, China (Figure 1). Of relatively small size, these parks were located in the high-density established districts of Shanghai, and they mainly served people in proximity. Of the 56 neighborhood parks less than 5 ha in Shanghai, those with an area between 1 ha and 2 ha accounted for the highest proportion (39.29%) (1 ha = 10,000 m^2^). The areas of the three selected neighborhood parks were 1.43 ha, 1.37 ha, and 1.42 ha, respectively, which were close to each other and represented ordinary neighborhood parks in Shanghai. The neighborhood parks were all constructed in the 1980s or 1990s, thus shared almost the same historical backgrounds, standard of construction, materials, and techniques [55].

**Figure 2:**
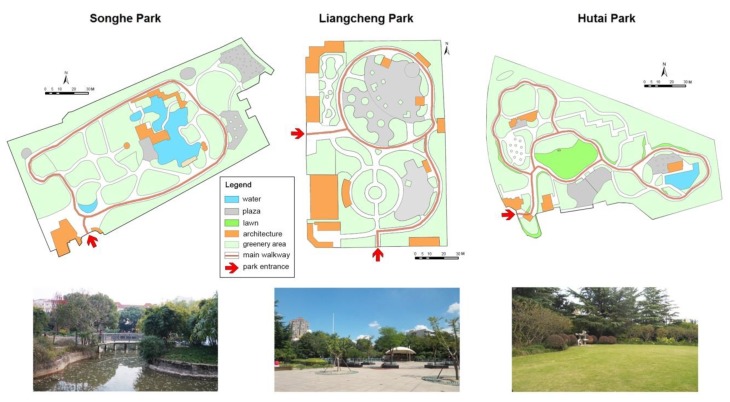
The plan and photo of the three neighborhood parks, with distinct features of water, plaza, and lawn respectively.

Although the parks were of similar size, they differed in landscape composition and spatial patterns. As shown in Figure 2, the landscape features in the parks were distinct from each other: Songhe Park had water features, much more than the other two parks; Liangcheng Park had a large plaza area, taking up 18.95% of the park area, which was nearly three times the one in Songhe Park and over two times the one in Hutai Park; Hutai Park had an area of lawn in the middle and sparsely decorated lawns along the walkways, while the other two parks had no lawn areas. Liangcheng Park was more accessible than the other two parks, as it had three gateways, while both Songhe Park and Hutai Park had only one entrance and exit. The three neighborhood parks had different walking routes—the main walkway in Songhe Park was a loop, while in Liangcheng Park it was a circle in the north and a curve in the south; in Hutai Park, three loops were connected together. Greenery refers to the site covered with trees, lawns, shrubs, or other plants. The greenery area except lawn is shown in light green in Figure 2. Songhe Park and Hutai Park had similar open spaces of greenery, which were more enclosed than the green space in Liangcheng Park.

### 2.2. Behavior Settings in the Parks

Specific environmental components in parks create particular behavior settings for physical activity, and this allows a primary focus on the relationship between design and use of neighborhood parks. The selected parks had different types of behavior settings formed by different landscape features, such as water, plaza, and lawn. As architecture is an integral part of the neighborhood park for leisure-time activity, architecture setting was also included in the study. The definitions for the behavior settings are presented in Table 1. It is worth noting that water settings refer to the areas near a water body, as water is mainly used for viewing in neighborhood parks, and playing in water is not allowed. Architecture settings refer specifically to the sites in landscape architectures providing an open view, so architectural amenities which were designed primarily for indoor use and weakly associated with the outdoor environments were excluded from the study.

**Figure 3:**
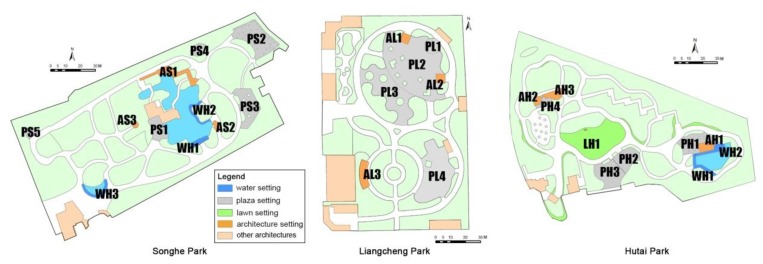
Codes of the behavior settings in parks. The first capital letter of the code is the abbreviation for the setting, and the second capital letter is the abbreviation for the park.

The mix of setting types was different in the parks. As shown in Figure 3 and Table 2, Songhe Park included three water settings, five plaza settings, and three architecture settings; Liangcheng Park included four plaza settings and three architecture settings; and Hutai Park included two water settings, four plaza settings, one lawn setting, and three architecture settings. Landscape architectures such as pavilions and pergolas were examined as architecture settings, but other architectures mainly for indoor use, such as toilet, café, restaurant, store, and management office, were not analyzed in this study (shown in light orange in Figure 3). The boundary of a setting is usually defined by material lines on the ground between different landscape components, such as the edge of a walkway or boundary of a structure [39].

Even behavior settings of the same type may differ in landscape attributes. In this study, behavior settings with different attributes were also considered to explore the environment–behavior relationship. All three neighborhood parks had plaza settings, but they were of different sizes. Landscape attributes such as the shade condition provided by tall trees and whether fitness or playground equipment was installed in the plaza varied among the settings. The plazas in parks were further categorized based on their size, shade of trees, and exercise equipment provided (Table 3). According to the plaza area, the settings were categorized into three groups: small (<100 m^2^), medium (100–500 m^2^), and large (>500 m^2^). Similarly for the architecture settings, they were categorized into two groups by area: small (<50 m^2^) and large (≥50 m^2^).

### 2.3. Data Collection

The behavior mapping method was used to explore visitors’ behaviors, their activity level, and the environmental context where the physical activities occurred [43,44,58]. The first phase involved field measurements to collect the detailed maps of the neighborhood parks, as a few parts of the parks were reconstructed over the years and no recent plans were found. Initial site observations were conducted in each park to identify the observing zones with the standing points based on the following rule: when the observer is standing at the location, all site conditions of the zone can be watched clearly, while the place is not very obvious to disturb the park’s visitors. The observing zones and standing points facilitated the observer’s work of recording in sequence the use of the park by people. A total of 26 observing zones covering all three parks were identified: nine in Songhe Park, eight in Liangcheng Park, and nine in Hutai Park. Within each zone, one standing point was selected. Because more people visited the parks in the morning than at noon, the duration at each standing point was set for 10 min as tested during the peak and off-peak hours. During the 10 min period, the observer scanned the observation zone one time visually from left to right, and the location of an individual was noted as a dot on the paper maps of the sites (1:500 scale), together with the codes representing the gender, age groups, and the activities. After the scan, the observer waited 10 min before moving to the next target zone, to make sure every round of observation was time comparable. Observations were conducted in all zones throughout the neighborhood park, and in this way the recorder could finish the scan of a neighborhood park within one and a half hours.

Systematic observations of the selected neighborhood parks were made in September 2015. September is early autumn in Shanghai; as the temperature is cooling down, it is great weather for outdoor activities. To analyze the daily use of neighborhood parks, data were gathered three times a day during weekdays of no rain (morning at 7:30 am–9:00 am, noon at 11:30 am–1:00 pm, and afternoon at 3:30 pm–5:00 pm). All three neighborhood parks (26 zones) were observed three times for each time period, resulting in 27 times of park scans (234 rounds of zone observations) in total.

### 2.4. Data Analysis

The hand-recorded data were used to create the spatial graphics and attribute tables in the geographic information system software ArcGIS 10 (Esri, Redlands, CA, USA). The types of activities were classified into three classes according to the activity level: sedentary, walk, and MVPA [43]. Each dot represented one person observed during the observation session and their level of physical activity (Appendix A). Descriptive statistical analysis was used to analyze physical activity in neighborhood parks and behavior settings, at different times of the day, and by age and gender. A two-way chi-squared test was conducted to examine the relationship between behavior settings (water, plaza, lawn, and architecture) and the frequency counts of physical activity for sedentary, walk, and MVPA. The association between the different attributes of the same setting (e.g., plazas of different size, shade condition, and exercise equipment, see Table 3) and the levels of physical activity was also examined using the chi-squared test. Cramér’s V is an effect size measurement for the chi-squared test of independence (0.1 for small, 0.3 for medium, and 0.5 for large) [59].

## 3. Results

### 3.1. Overall Physical Activity Levels in Neighborhood Parks

**Figure 4:**
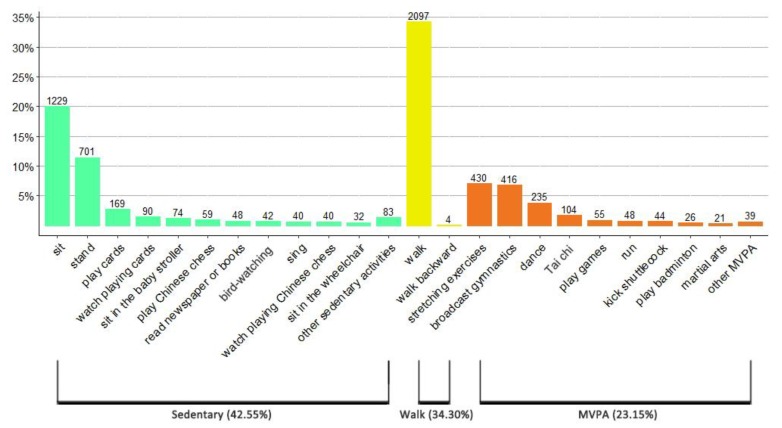
Observed predominant activities (in which over 30 people participated) by physical activity levels.

**Figure 5:**
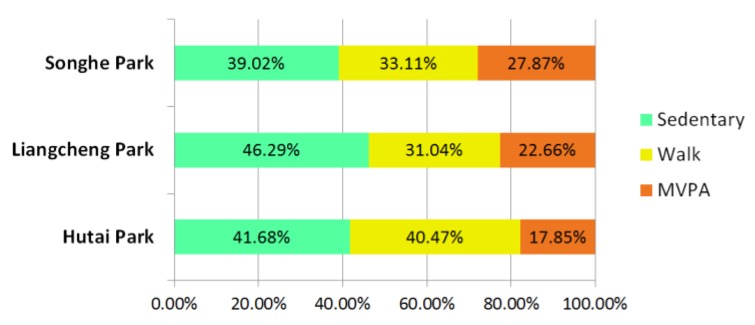
Percentage of the observed park users participating in the different levels of physical activity.

For all three neighborhood parks, a total of 6126 observations were documented, belonging to a list of 37 activities (Table 4). Overall, visitors engaged in more sedentary activity (42.55%) than walk (34.30%) and MVPA (23.15%) in the neighborhood parks (Figure 4). The levels of physical activity of park users varied among the three parks, but shared the same order: sedentary activity was mostly observed, walk activity was less, and MVPA was the least (Figure 5).

From the mapped data (Appendix A), the majority of sedentary activity was observed at the entrance, around the recreational amenity, in the landscape architecture, on the edge of the plaza, and along the walkway. The walking people were mostly coded along the main walkway and some of the narrow walkways. The observed MVPA participants were gathered in the plaza, on the lawn, in the grove, and along the walkway. The mostly commonly observed MVPAs were stretching exercises, broadcast gymnastics, dancing, and practicing Tai chi.

### 3.2. Physical Activity Levels by Setting Types

**Figure 6:**
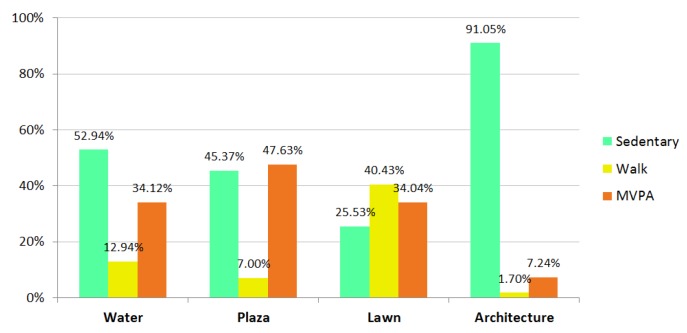
Percentage of observed park users by different landscape settings.

The majority of total activity observations were distributed across four types of behavior setting: water, plaza, lawn, and architecture (Appendix A). Results of the two-way chi-squared test revealed that levels of physical activity varied with the different types of behavior settings: water, plaza, lawn, and architecture (χ^2^ = 543.90, *p* < 0.001), at a medium effect size (Cramér’s V = 0.313). As shown in Table 5 and Figure 6, people were more likely to be sedentary in the architecture setting (91.05%); more people engaged in walk activity on the lawn (40.43%); and more people were involved in MVPA in the plaza (47.63%).

### 3.3. Physical Activity Levels by Setting Attributes

Differences in levels of physical activity were created by different types of behavior settings and by different attributes of the same type of the behavior setting. In total, there were thirteen plaza settings in the parks, which varied in area, shade of trees, and exercise equipment (Table 3). The chi-squared test was used to test for independence of plaza size (small, medium, and large) and levels of physical activity (sedentary, walk, and MVPA). A significant relationship was found between the size categories of plaza and levels of physical activity (χ^2^ = 71.671, *p* < 0.001, Cramér’s V = 0.136) (Table 6). People were more likely to participate in MVPA on the medium-sized plaza than on the large one (55.37% compared to 39.76%); people on the large plaza were more likely to be sedentary than on the medium-sized plaza (54.12% compared to 35.58%). Slightly more people in MVPA were observed in small plazas than expected, but most activities were conducted in small groups due to limited space.

The amount of use and level of activity were also affected by the shade of trees in the plaza, that people preferred to conduct MVPA in the plazas with taller trees providing abundant shade (χ^2^ = 31.87, *p* < 0.001, Cramér’s V = 0.128). Plazas with fitness or playground equipment attracted more people engaged in MVPA than those without (54.19% compared to 43.17%), and the difference was significant (χ^2^ = 27.70, *p* < 0.001, Cramér’s V = 0.119).

Big architecture (≥50 m^2^) attracted more people involved in MVPA than the small architecture setting (<50 m^2^) (9.76% compared to 2.47%), and a significant relationship was found between size category and level of activity (χ^2^ = 16.82, *p* < 0.001, Cramér’s V = 0.155).

### 3.4. Other Affecting Factors for Levels of Physical Activity

#### 3.4.1. Times of the Day

**Figure 7:**
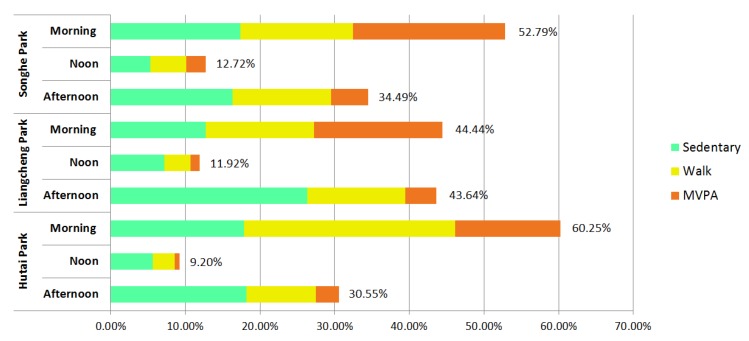
Percentage of park use by different time periods of the day.

The numbers of people observed and their physical activity levels differed during the three time periods of the day (Figure 7). Overall, more people visited the neighborhood parks in the morning (52.49%) than in the afternoon (36.23%), and much fewer people visited the parks during the noon time. People were more likely to be active in the morning than other time periods: the average proportion of people involved in MVPA was 17.22% in the morning (vs. 1.50% at noon and 4.07% in the afternoon) for the three neighborhood parks.

#### 3.4.2. Age and Gender Differences

**Figure 8:**
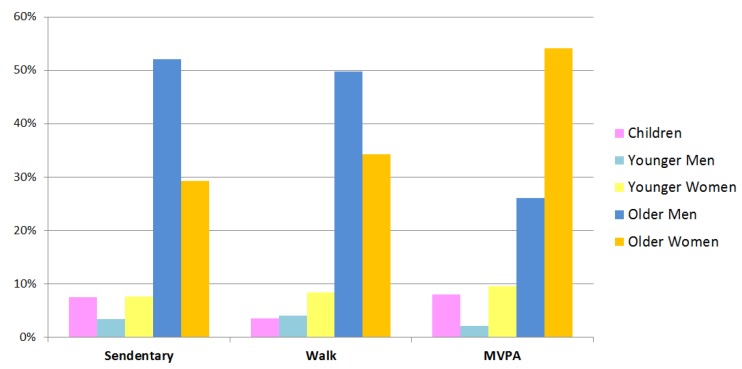
Percentage of observed park use by different age and gender groups.

As shown in Figure 8, over 50% of the park visitors in sedentary status were older men, much more than the other age groups. More numbers of older men involved in walk activity (49.79%), while a lot more older women participated in MVPA (54.16%).

## 4. Discussions

### 4.1. Setting Types and Physical Activity Level

This study investigated the association between specific behavior settings and users’ level of physical activity in neighborhood parks. Observations revealed that park visitors participated in different levels of activity in various types of settings, such as water, plaza, lawn, and architecture. People were more likely to be physically active in the plaza setting, be involved in walk activity on the lawn, and be sedentary in the landscape architecture setting. In addition, it was noted that behavior settings of the same type but with different landscape attributes (e.g., size, shade of trees, and exercise equipment) differed significantly in the levels of activity that people were involved in.

Water setting. People generally had lower levels of activity by the water, but differences were found among the settings. Of the five water settings, a greater averaged percentage of MVPA participation were observed in water settings WS2 and WH1 than in the other three water settings (53.85% compared to 21.59%). This may due to their different site locations, and the settings away from the main walkway can provide more privacy for activity.

Plaza setting. The highest MVPA level was observed in plaza settings. Medium-sized plazas with shading trees and exercise equipment attracted more MVPA participants. In the plaza, people exercised with fitness equipment (e.g., PS2, PS3, and PH2) or used the paved areas to dance and conduct broadcast gymnastics (e.g., PL1, PL2, and PH1). People on the edge of plazas were attracted to face in the direction of the human activity, supporting the previous design theory of “to see and be seen” [60]. In small plazas surrounded by buildings (e.g., PS1, PS4, and PH4), traditional exercise such as playing Chinese swords or practicing Tai chi or martial arts occurred, as these places were relatively quiet and suitable for people who wanted to exercise alone or in small groups.

Lawn setting. More proportions of walking people were observed on the lawn than in other settings. The lawn of Hutai Park was an important site for children walking, running, and playing games, accompanied with their parents, and also attracted the attention of passersby.

Architecture setting. Recreational amenities such as pavilions and pergolas attracted people gathering together to do sedentary activities, including playing cards and Chinese chess. It is worth noting that people were involved in different sedentary activities during different times of the day. For example, at one pavilion in Liangcheng Park, many people were observed sitting and drinking tea in the morning, but playing Chinese chess or watching others play in the afternoon.

Based on the behavior mapping data, it can also be speculated that the walkway structure affected the use by walkers. A larger proportion of walkers was coded in Hutai Park (40.47%) than in Songhe Park (33.11%) and Liangcheng Park (31.04%). It may be because the combined looped walkway with a systematic structure promoted walk activity. This finding was supported by previous studies showing walking loops can increase users’ walk activity [61,62].

This observational study provides useful information for improving the amount and intensity of physical activity in neighborhood parks. The results generally supported previous research and theory suggesting that landscape attributes can influence park use and physical activity [33,35]. The study also demonstrated that behavior mapping is a useful tool for objectively measuring the relationship between park features and visitors’ physical activity [39,43,46].

### 4.2. Cultural Differences in Park Use

This study provided clues about the differences in park visitors’ behaviors between China and Western countries. Activities such as performing broadcast gymnastics, doing stretching exercises, and playing Chinese chess were commonly observed in Chinese park settings, but not in European parks and American parks, where playing basketball, football, or tennis and walking a dog occurred more often [21,45]. Women tended to be more physically active than men in Shanghai neighborhood parks, which is contrary to the results found in an American study [63]. The possible reasons may be that some activities, such as plaza dancing and broadcast gymnastics, are popular with middle-aged and retired women in China, but are less popular among men. On the other hand, although men generally prefer ball games more than women, the settings in neighborhood parks cannot support (i.e., afford) this type of activity, due to limited space, flow of people, and lack of facilities. Park management and designers could consider the cultural differences in park use, and provide more space and facilities for those less likely to be active.

### 4.3. Strengths and Limitations

Using the behavior mapping method, this study addressed the specific landscape features associated with intensity of activity in neighborhood parks. This may help designers and policy makers understand the links between physical activity and outdoor design.

While behavior mapping proved to be an effective way to record the location of individuals, their characteristics, and their physical activity, it is time consuming and may not be suitable for monitoring park use in a large area, especially when the data are initially hand-recorded on the sites. Future studies could combine this method with other techniques such as video-taping and unmanned aerial vehicles, to make the process of data collection more precise and efficient [64].

Although this study took setting types (e.g., water, plaza, lawn, and architecture) into consideration when analyzing the use patterns of neighborhood parks, other landscape characteristics may also influence park usage, such as facilities, amenities, and plantings [31]. Due to time constraints, this study only collected data from three neighborhood parks of Shanghai, and only in the autumn. Future research could conduct similar work in urban parks of different sizes and with various landscape characteristics, during the four seasons and all day long (from morning till night), so more detailed information could be collected to explore the impacts of different landscape features on users’ behaviors.

## 5. Conclusions

As a vital component of the urban green space system, neighborhood parks play a critical role in promoting physical activity. By examining the association between behavior settings and physical activity, this study provides a preliminary understanding of the actual use of neighborhood parks in Shanghai, China, and thus can help local landscape architects design active outdoor environments to increase the physical activity levels of park users. It is also possible that the findings of this study can be compared with those of other research, to detect the effects of cultural factors on space–behavior relationships.

## Figures and Tables

**Table 1:** Definitions for the behavior settings.

Setting Type	Definition
Water	The accessible sites by the waterfront, where people’s activity is directly related to the water body.
Plaza	The hardscape feature in the park suitable for gathering and activity.
Lawn	The green space covered with grass, and people can step on it.
Architecture	The sites in landscape architectures, namely pavilions and pergolas, where people can enjoy an open view in an outdoor living space.

**Table 2:** List of behavior settings in the three neighborhood parks.

Setting Type	Number of Behavior Settings		
	Songhe Park	Liangcheng Park	Hutai Park
Water	3 (WS1–WS3)	0	2 (WH1–WH2)
Plaza	5 (PS1–PS5)	4 (PL1–PL4)	4 (PH1–PH4)
Lawn	0	0	1 (LH1)
Architecture	3 (AS1–AS3)	3 (AL1–AL3)	3 (AH1–AH3)

Note: Codes of the behavior settings are shown in parentheses.

**Table 3:** Attributes of plaza settings in the parks.

Plaza Code	Area (m^2^)	Size Category	Shade Trees	Exercise Equipment
PS1	132.78	Medium	Plenty	No
PS2	400.53	Medium	Plenty	Yes
PS3	271.72	Medium	Scarce	Yes
PS4	71.88	Small	Plenty	No
PS5	38.29	Small	Plenty	No
PL1	88.69	Small	Scarce	No
PL2	1237.50	Large	Scarce	No
PL3	652.43	Large	Plenty	No
PL4	729.59	Large	Scarce	Yes
PH1	238.19	Medium	Scarce	No
PH2	198.41	Medium	Scarce	No
PH3	501.79	Large	Scarce	No
PH4	45.62	Small	Plenty	No

**Table 4:** Physical activities occurring in the neighborhood parks (numbers of people observed).

Activity Levels	Types of Physical Activity
Sedentary	sit (1229), stand (701), play cards (169), watch playing cards (90), sit in the baby stroller (74), play Chinese chess (59), read newspaper or books (48), bird-watching (42), sing (40), watch playing Chinese chess (40), sit in the wheelchair (32), take photos (16), use phones (14), eat food (12), listen to radio or music (9), lay on a bench (8), knit (8), embroider (7), play musical instruments (5), trim vegetables for cooking (4)
Walk	walk (2097), walk backward (4)
MVPA	stretching exercises (430), broadcast gymnastics (416), dance (235), Tai chi (104), play games (55), run (48), kick shuttlecock (44), play badminton (26), martial arts (21), roller skate (12), play Chinese swords (10), ride kids’ bikes (9), kick balls (4), play with water (3), fly kites (1)

**Table 5:** Cross tabulation of setting type and level of physical activity.

Setting Type	Level of Physical Activity		
	Sedentary	Walk	MVPA
Water	45 (−0.7)	11 (2.5)	29 (−0.5)
Plaza	882 (−18.6)	136 (1.9)	926 (18.1)
Lawn	12 (−4.4)	19 (9.6)	16 (−0.4)
Architecture	641 (21.2)	12 (−5.9)	51 (−18.8)

Note: Adjusted residuals appear in parentheses beside observed frequencies.

**Table 6:** Cross tabulation of plaza size and level of physical activity.

Plaza Size	Level of Physical Activity			
	Sedentary	Walk	MVPA	Total
*Small size (<100 m* ^2^ *)*				
Count	68	5	102	175
Percentage within plaza size	38.85%	2.86%	58.29%	100%
Adjusted residuals	−1.8	−2.3	3.0	
				
*Medium size (100–500 m* ^2^ *)*				
Count	275	70	428	773
Percentage within plaza size	35.58%	9.06%	55.37%	100%
Adjusted residuals	−7.0	2.9	5.5	
				
*Large size (>500 m* ^2^ *)*				
Count	539	61	396	996
Percentage within plaza size	54.12%	6.12%	39.76%	100%
Adjusted residuals	7.9	−1.5	−7.1	

**Figure A1:**
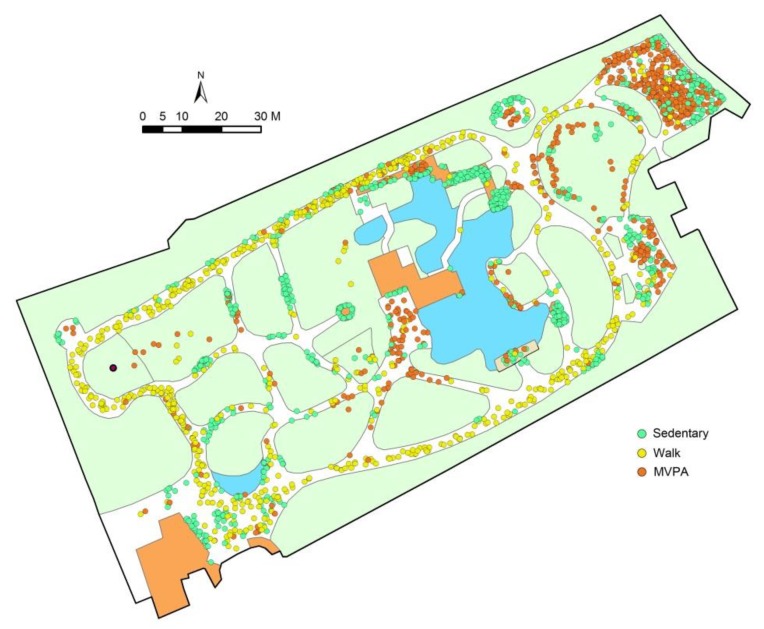
Behavior map of Songhe Park showing the distribution of users (the data were from nine rounds of observations: morning, noon and afternoon, three times each).

**Figure A2:**
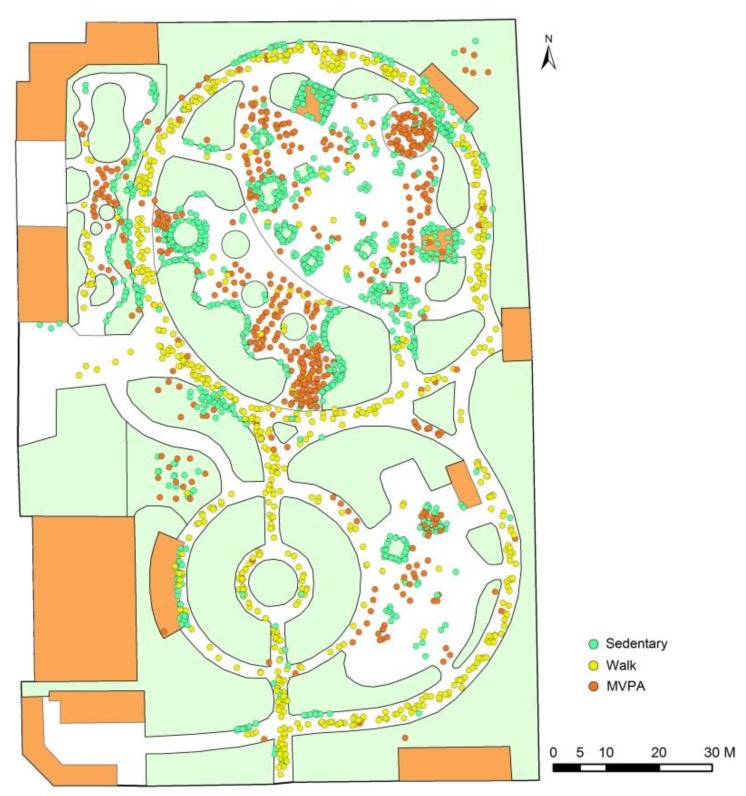
Behavior map of Liangcheng Park showing the distribution of users (the data were from nine rounds of observations: morning, noon and afternoon, three times each).

**Figure A3:**
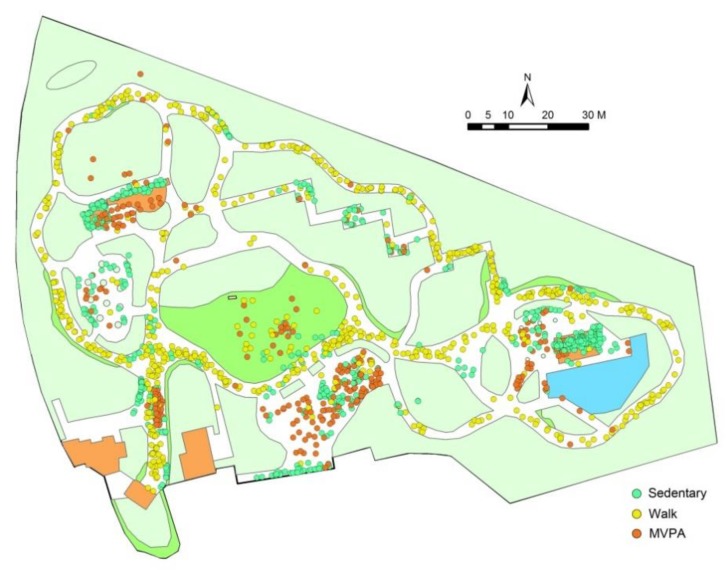
Behavior map of Hutai Park showing the distribution of users (the data were from nine rounds of observations: morning, noon and afternoon, three times each).

## References

[1] Churilla J.R., Zoeller R.F. (2008). Physical activity: Physical activity and the metabolic syndrome: A review of the evidence. Am. J. Lifestyle Med..

[2] Hallal P.C., Victora C.G., Azevedo M.R., Wells J.C. (2006). Adolescent physical activity and health: A systematic review. Sports Med..

[3] Warburton D.E.R., Nicol C.W., Bredin S.S.D. (2006). Health benefits of physical activity: The evidence. Can. Med Assoc. J..

[4] Carlson S.A., Adams E.K., Yang Z., Fulton J.E. (2018). Percentage of deaths associated with inadequate physical activity in the United States. Prev. Chronic Dis..

[5] Centre for Economics and Business Research The Economic Cost of Physical Inactivity in Europe. https://inactivity-time-bomb.nowwemove.com/report/.

[6] Ng S.W., Popkin B.M. (2012). Time use and physical activity: A shift away from movement across the globe. Obes. Rev..

[7] Muntner P., Gu D., Wildman R.P., Chen J., Qan W., Whelton P.K., He J. (2005). Prevalence of physical activity among Chinese adults: Results from the international collaborative study of cardiovascular disease in Asia. Am. J. Public Health.

[8] Zhang J., Chaaban J. (2013). The economic cost of physical inactivity in China. Prev. Med..

[9] Wu S., Luo Y., Qiu X., Bao M. (2017). Building a healthy China by enhancing physical activity: Priorities, challenges, and strategies. J. Sport Health Sci..

[10] Lee C., Moudon A.V. (2008). Neighbourhood design and physical activity. Build. Res. Inf..

[11] Saelens B.E., Handy S.L. (2008). Built environment correlates of walking: A review. Med. Sci. Sports Exerc..

[12] Coombes E., Jones A.P., Hillsdon M. (2010). The relationship of physical activity and overweight to objectively measured green space accessibility and use. Soc. Sci. Med..

[13] Koohsari M.J., Karakiewicz J.A., Kaczynski A.T. (2012). Public open space and walking: The role of proximity, perceptual qualities of the surrounding built environment, and street configuration. Environ. Behav..

[14] Lachowycz K., Jones A.P. (2011). Greenspace and obesity: A systematic review of the evidence. Obes. Rev..

[15] Shin W.-H., Kweon B.-S., Shin W.-J. (2011). The distance effects of environmental variables on older African American women’s physical activity in Texas. Landsc. Urban Plan..

[16] Rogerson M., Gladwell V.F., Gallagher D.J., Barton J.L. (2016). Influences of green outdoors versus indoors environmental settings on psychological and social outcomes of controlled exercise. Int. J. Environ. Res. Public Health.

[17] Hartig T., Mitchell R., de Vries S., Frumkin H. (2014). Nature and health. Annu. Rev. Public Health.

[18] Kemperman A., Timmermans H. (2014). Green spaces in the direct living environment and social contacts of the aging population. Landsc. Urban Plan..

[19] Cohen D.A., Kristin J.L. (2018). How Can Neighborhood Parks Be Used to Increase Physical Activity?.

[20] Berman M.G., Jonides J., Kaplan S. (2008). The cognitive benefits of interacting with nature. Psychol. Sci..

[21] Cohen D.A., Han B., Nagel C.J., Harnik P., McKenzie T.L., Evenson K.R., Marsh T., Williamson S., Vaughan C., Katta S. (2016). The first national study of neighborhood parks: Implications for physical activity. Am. J. Prev. Med..

[22] Mennis J., Mason M., Ambrus A. (2018). Urban greenspace is associated with reduced psychological stress among adolescents: A Geographic Ecological Momentary Assessment (GEMA) analysis of activity space. Landsc. Urban Plan..

[23] Sugiyama T., Carver A., Koohsari M.J., Veitch J. (2018). Advantages of public green spaces in enhancing population health. Landsc. Urban Plan..

[24] Tinsley H.E.A., Tinsley D.J., Croskeys C.E. (2002). Park usage, social milieu, and psychosocial benefits of park use reported by older urban park users from four ethnic groups. Leis. Sci..

[25] Veitch J., Salmon J., Parker K., Bangay S., Deforche B., Timperio A. (2016). Adolescents’ ratings of features of parks that encourage park visitation and physical activity. Int. J. Behav. Nutr. Phys. Act..

[26] Kowaleski-Jones L., Fan J.X., Wen M., Hanson H. (2017). Neighborhood context and youth physical activity: Differential associations by gender and age. Am. J. Health Promot..

[27] Zhang W., Yang J., Ma L., Huang C. (2015). Factors affecting the use of urban green spaces for physical activities: Views of young urban residents in Beijing. Urban For. Urban Green..

[28] Baran P.K., Smith W.R., Moore R.C., Floyd M.F., Bocarro J.N., Cosco N.G., Danninger T.M. (2014). Park use among youth and adults: Examination of individual, social, and urban form factors. Environ. Behav..

[29] Koohsari M.J., Mavoa S., Villanueva K., Sugiyama T., Badland H., Kaczynski A.T., Owen N., Giles-Corti B. (2015). Public open space, physical activity, urban design and public health: Concepts, methods and research agenda. Health Place.

[30] Park K. (2019). Park and neighborhood attributes associated with park use: An observational study using unmanned aerial vehicles. Environ. Behav..

[31] Costigan S.A., Veitch J., Crawford D., Carver A., Timperio A. (2017). A Cross-sectional investigation of the importance of park features for promoting regular physical activity in parks. Int. J. Environ. Res. Public Health.

[32] Kaczynski A.T., Potwarka L.R., Saelens B.E. (2008). Association of park size, distance, and features with physical activity in neighborhood parks. Am. J. Public Health.

[33] Van Hecke L., Ghekiere A., Veitch J., Van Dyck D., Van Cauwenberg J., Clarys P., Deforche B. (2018). Public open space characteristics influencing adolescents’ use and physical activity: A systematic literature review of qualitative and quantitative studies. Health Place.

[34] Zacharias J., Stathopoulos T., Wu H. (2004). Spatial behavior in San Francisco’s plazas: The effects of microclimate, other people, and environmental design. Environ. Behav..

[35] McCormack G.R., Rock M., Toohey A.M., Hignell D. (2010). Characteristics of urban parks associated with park use and physical activity: A review of qualitative research. Health Place.

[36] Peschardt K.K., Stigsdotter U.K. (2013). Associations between park characteristics and perceived restorativeness of small public urban green spaces. Landsc. Urban Plan..

[37] Veitch J., Salmon J., Deforche B., Ghekiere A., Van Cauwenberg J., Bangay S., Timperio A. (2017). Park attributes that encourage park visitation among adolescents: A conjoint analysis. Landsc. Urban Plan..

[38] Hill M.R. (1984). Stalking the urban pedestrian: A comparison of questionnaire and tracking methodologies for behavioral mapping in large-scale environments. Environ. Behav..

[39] Cosco N.G., Moore R.C., Islam M.Z. (2010). Behavior mapping: A method for linking preschool physical activity and outdoor design. Med. Sci. Sports Exerc..

[40] McKenzie T.L., Marshall S.J., Sallis J.F., Conway T.L. (2000). Leisure-time physical activity in school environments: An observational study using SOPLAY. Prev. Med..

[41] Blennerhassett J.M., Borschmann K.N., Lipson-Smith R.A., Bernhardt J. (2018). Behavioral mapping of patient activity to explore the built environment during rehabilitation. HERD Health Environ. Res. Des. J..

[42] Hamilton K., Kaczynski A.T., Fair M.L., Lévesque L. (2017). Examining the relationship between park neighborhoods, features, cleanliness, and condition with observed weekday park usage and physical activity: A case study. J. Environ. Public Health.

[43] McKenzie T.L., Cohen D.A., Sehgal A., Williamson S., Golinelli D. (2006). System for Observing Play and Recreation in Communities (SOPARC): Reliability and feasibility measures. J. Phys. Act. Health.

[44] Evenson K.R., Jones S.A., Holliday K.M., Cohen D.A., McKenzie T.L. (2016). Park characteristics, use, and physical activity: A review of studies using SOPARC (System for Observing Play and Recreation in Communities). Prev. Med..

[45] Goličnik B., Ward Thompson C. (2010). Emerging relationships between design and use of urban park spaces. Landsc. Urban Plan..

[46] Evenson K.R., Williamson S., Han B., McKenzie T.L., Cohen D.A. (2019). United States’ neighborhood park use and physical activity over two years: The national study of neighborhood parks. Prev. Med..

[47] Gibson J.J. (1979). The Ecological Approach to Visual Perception.

[48] Barker R.G. (1968). Ecological Psychology: Concepts and Methods for Studying the Environment of Human Behavior.

[49] Floyd M.F., Spengler J.O., Maddock J.E., Gobster P.H., Suau L. (2008). Environmental and social correlates of physical activity in neighborhood parks: An observational study in Tampa and Chicago. Leis. Sci..

[50] Xiao Y., Wang D., Fang J. (2019). Exploring the disparities in park access through mobile phone data: Evidence from Shanghai, China. Landsc. Urban Plan..

[51] Xiao Y., Wang Z., Li Z., Tang Z. (2017). An assessment of urban park access in Shanghai—Implications for the social equity in urban China. Landsc. Urban Plan..

[52] Nordh H., Hartig T., Hagerhall C.M., Fry G. (2009). Components of small urban parks that predict the possibility for restoration. Urban For. Urban Green..

[53] Wang X., Rodiek S., Wu C., Chen Y., Li Y. (2016). Stress recovery and restorative effects of viewing different urban park scenes in Shanghai, China. Urban For. Urban Green..

[54] Zhai Y., Baran P.K., Wu C. (2018). Spatial distributions and use patterns of user groups in urban forest parks: An examination utilizing GPS tracker. Urban For. Urban Green..

[55] Shanghai Landscaping & City Appearance Administrative Bureau. http://lhsr.sh.gov.cn/sites/ShanghaiGreen/dyn/ViewIndex.ashx.

[56] The State Council, The People’s Republic of China http://www.gov.cn/xhtml/2016gov/images/guoqing/bigmap.jpg.

[57] Google Maps. http://maps.google.com.

[58] Cohen D.A., Setodji C., Evenson K.R., Ward P., Lapham S., Hillier A., McKenzie T.L. (2011). How much observation is enough? Refining the administration of SOPARC. J. Phys. Act. Health.

[59] Abbott M.L., McKinney J. (2013). Understanding and Applying Research Design.

[60] Rutledge A.J. (1985). A Visual Approach to Park Design (Garland Series in Design).

[61] Arnold C. (2017). Exercise bargain: Are walking loops worth the investment?. Environ. Health Perspect..

[62] Cohen D.A., Han B., Evenson K.R., Nagel C., McKenzie T.L., Marsh T., Williamson S., Harnik P. (2017). The Prevalence and Use of Walking Loops in Neighborhood Parks: A National Study. Environ. Health Perspect..

[63] Derose K.P., Han B., Williamson S., Cohen D.A. (2018). Gender Disparities in Park Use and Physical Activity among Residents of High-Poverty Neighborhoods in Los Angeles. Women Health Issues.

[64] Park K., Ewing R. (2017). The usability of unmanned aerial vehicles (UAVs) for measuring park-based physical activity. Landsc. Urban Plan..

